# Bulletin of the Buffalo General Hospital

**Published:** 1912-07

**Authors:** 


**Bulletin of the Buffalo General Hospital**

VOL. II. JULY, 1912 No. I.

### The Board of Trustees

AT the April meeting of the Board of Trustees, Mr. George R. Howard was elected president, to fill the vacancy caused by Mr. Pardee's death, and Mr. Carlton M. Smith was made vice-president. At the same meeting Mrs. Pardee was made a trustee.

Among the committees appointed during the past three months is one to consider the proper commemoration of gifts to the hospital and notable services rendered it. This is a point upon which there is need of action, and upon which the procedure of other hospitals throws little light. The endowed bed and endowed room are everywhere established, but at this point the hospital imagination wavers—perhaps through lack of occasion for wider flights. For with the exception of a few institutions in old communities, our American hospitals are poorly endowed; and they can make today a double plea for public support, for they supply generously the remedial needs of the moment, and they are centers of preventive work in their communities.

The trustees hope to work out a general scheme for making appropriate recognition of the varied gifts of money and service which have built up the General Hospital from the tiny beginning of more than fifty years ago. A memorial tablet to Mr. Pardee has already been voted.

The department of social service has also been under consideration. Its work is growing in scope and efficiency, and the question of its support is a serious one, as the resources of the Hospital do not permit any substantial diversion of funds to its needs. A fuller statement of its work will be found elsewhere in the Bulletin.

Under authorization from the trustees, the General Hospital cooperated with the Homeopathic and Children's Hospitals in a display at the recent Child's Welfare Exhibit. Nurses from the three institutions were in attendance in turn during the exhibition hours. The General Hospital showed a series of photographs of the Harrington and of groups of its inmates, and there was also a pictorial representation, doll-house size, showing the superiority of hospital care for the sick child over that given him at home. The invalid doll lay in a squalid room, which it shared with a number of other dolls, actively employed, and with two dogs, of truly terrifying size in comparison with the human members of the family group. An adjoining compartment showed the invalid in a hospital cot, attended by an immaculate nurse. The moral could be read by those who ran: it was "better" at the hospital, but more sociable at home.

### Superintendent of Nurses

The resignation of Miss Rye Morley as Superintendent of Nurses, which the Hospital administration accepted with much regret, went into effect July 1st. Miss Morley has been at the General Hospital for four years—two years as first Assistant Superintendent of Nurses, and for the last two years at the head of the Training School. In September she takes the corresponding position at Mt. Sinai Hospital in New York, an institution with accommodations for five hundred patients, and a school of one hundred and seventy-four nurses. Miss Morley was recently appointed a member of the State Examining Board for Nurses, but declined the appointment.

Her successor at the Buffalo General Hospital is Mrs. Mabel McDonald, a graduate of the Hospital's training school, who has filled the exacting position of Night Supervisor of Nurses there for the past year.

The Hospital may well look with satisfaction upon the number of its graduates who are filling responsible administrative positions. Including such positions at the General Hospital itself, twelve of its graduates are engaged in important institutional work in Buffalo; six are superintendents of hospitals in smaller cities, five are in New York hospitals, and four in Brooklyn—two of these four as assistant superintendents.

### Gifts

At the April meeting of the Board of Trustees a check of $10,000 was received from Mrs. Pardee, as a gift from Mr. Pardee, endowing one of the semi-private rooms. This establishes a new class of endowments at the hospital, and is a most fitting memorial of Mr. Pardee, as he felt that the semi-private rooms met the needs of a class of patients very inadequaely provided for by our hospitals.

Another free bed has been provided in the maternity service at the Harrington, through the generosity of Mrs. W. Austin Wadsworth of Geneseo.

The bequest of $1,000 from Mr. William Pryor Letchworth has been received. It is to be added to the general endowment fund.

**Commencement**

The graduating exercises of the Training School were held, as usual, on the twelfth of June, in the Nurses' Home. The opening prayer was by the Rev. W. Hamilton Benham, and the address of the evening by Mr. Mellen of the Lafayette High School. The diplomas were presented by Dr. Ward Plummer, and the badges of the hospital by Mr. Howard, president of the Board of Trustees. The music for the exercises themselves was furnished by the choir of Westminster Church, and for the reception afterwards by Kuhn's orchestra. The usual dance was omitted, out of respect for Mr. Pardee's memory.

The class this year numbers eighteen members, half of whom are Canadians. This was the percentage in last year's class also.

For the most part, this year's graduates intend to devote themselves to private nursing; one, however, enters the School of Health and Nursing at Columbia University, to prepare herself for teaching. A second, also, may take up teaching later.

**The Social Service Work**

The most recent development in the social service work has been its concentration into three general divisions—the work for convalescents, the child welfare work, and that in connection with the maternity ward. Each division is under the special supervision of one worker, and there is no question but that more ground is covered and better work done by such specialization. The result is shown in eight or nine cases in the maternity ward in place of the former two or three, and of double the number of children's cases. The plan is to extend the work by the help of volunteer workers, under special supervision.

The work for convalescents consists, in the main, in prolonging the period of rest, either by utilizing Buffalo's scanty provision for such cases, or by securing country board for them; or sometimes a change of occupation may be required, or the finding of some light work for the handicapped. A most important work for patients leaving the hospital is often to keep them in touch with dispensaries or with private physicians, as their case demands. The patient who needs this sort of following up is not infrequently one with a high appreciation of what the hospital has done for him; after its magic touch, what more can be needed?

The child welfare work begins in the wards with kindergarten instruction, and often secures the temporary transfer of a child to a good boarding home, in cooperation with the Superintendent of the Poor's country department for children. Ignorant mothers are taught, at home, something of hygiene and proper feeding. The work in the maternity ward is perhaps the most urgent of all. Sometimes it takes the form of council and temporary relief, which enables a woman with a family to take advantage of the hospital care; sometimes legal aid must be secured for a deserted or unsupported wife. The unmarried mothers constitute a difficult and delicate class to deal with, often taxing heavily the resources of the workers. In the past year thirty-nine maternity cases were referred to the social service department.

During this beginning period, the District Nursing Association has been the staff upon which this department has leaned, and the social work can reach no degree of prosperity in which it will not need the Association's cooperation and its counsel.

For the furtherance of all the interests involved, an executive committee has been appointed, in accordance with the action of the Trustees, noted in the last Bulletin, one of whose duties shall be to reach the public interest, without which work of this kind cannot prosper. The support which it needs can be counted upon with confidence, when its intent and its scope are understood.

---

Plasmodial Anemia.—In spite of the modern theory of the etiology of malaria and malarial affections (mosquito-borne infection) this plasmodial disease continues to be rife in certain sections of the country and bids fair to be, like "the poor," "always with us."

Every physician of experience appreciates the principles which should guide him in the treatment of various acute manifestations of paludal poisoning, i. e., the destruction of the plasmodial hosts which have invaded the blood and which if not eliminated, consume and destroy the red cells, the vital element of the circulating fluid.

When this purpose has once been accomplished the patient is but partly cured; the damage done to the red corpuscles must be repaired and the vitality of the blood restored, if re-infection is to be avoided. If there is any one condition in which direct hematinic or blood-building therapy is positively indicated, it is in Post-Malarial Anemia. As soon as the febrile period has passed, iron, in some form, should be given in full dosage. Pepto-Mangan (Gude) constitutes the ideal method of administering this essential blood-building agent in this as well as in any anemic condition. Both the iron and manganese in Pepto-Mangan are in organic combination with peptones and are therefore easily and promptly absorbed and assimilated without causing digestive derangement or producing constipation.

